# Erosive Tooth Wear in Children and Adolescents: Awareness, Knowledge, and Management: A Case-Based Questionnaire Among Greek Dentists

**DOI:** 10.3390/dj13060249

**Published:** 2025-06-03

**Authors:** Sofia Chatzimarkou, Kyriaki Seremidi, William Papaioannou, Diana Mortensen, Svante Twetman, Sotiria Gizani

**Affiliations:** 1 Department of Paediatric Dentistry, School of Dentistry, National and Kapodistrian University of Athens, 2 Thivon Street, 115 27 Athens, Greece; 2 Department of Preventive and Community Dentistry, School of Dentistry, National and Kapodistrian University of Athens, 2 Thivon Street, 115 27 Athens, Greece; vpapaio@dent.uoa.gr; 3 Department of Odontology, Faculty of Health and Medical Sciences, University of Copenhagen, Nørre Alle’ 20, DK-2200 Copenhagen N, Denmark

**Keywords:** dental erosion, diet, questionnaire, treatment, clinical scoring, saliva

## Abstract

**Background/Objective**: With a prevalence linked to modern lifestyle, erosive tooth wear (ETW) is a growing clinical problem among children and adolescents. The aim of this cross-sectional study was to record the awareness and knowledge of ETW among Greek dentists and to explore their preferred treatment decisions. **Methods**: A case-based digital questionnaire was distributed to a stratified sample of dentists practising in Athens (n = 218). We collected data regarding clinical scoring, possible aetiological factors, and clinical management of ETW and used descriptive statistics, chi-square tests, and odds ratio calculations to process the outcome. **Results**: The response rate was 92%. The majority (71%) recorded ETW among their patients, but only 5% used an established and validated scoring system. Almost 1/3 registered only the location of the lesions. Over 70% disclosed the possible causes of ETW, with frequent consumption of fizzy soft drinks (67%) and acidic foods (56%) being the most common. Most respondents managed the ETW patients in their own clinic, while 23% referred them to another dentist or a university clinic. The respondents focused on secondary prevention (dietary advice, oral hygiene, and fluoride exposure) and preferred less invasive options for anterior teeth, with restorative care restricted to the lower molars. **Conclusions**: The majority of the dentists participating in this survey registered ETW and its possible causes and seem to have adopted a preventive and minimally invasive approach for the management in children and adolescents. For the case documentation, a minority took advantage of standardised scoring systems for lesions and dietary habits, and very few performed salivary diagnostics. The findings underscore the need of continuing education to offer updates on the most current guidelines and focusing on patients’ needs and expectations.

## 1. Introduction

Erosive tooth wear is a non-carious tooth surface loss, creating irreversible defects of the dental hard tissues. The condition initially affects enamel by decreasing its thickness and progresses thereafter into dentin due to chemical dissolution by acids of non-bacterial origin [1]. The aetiological factors are intrinsic and/or extrinsic; the intrinsic factors include gastroesophageal reflux disease (GERD), systemic diseases, medication, and eating disorders [2,3]. The extrinsic factors include consumption of acidic food and carbonated soft drinks, a vegan diet, and tooth bleaching [1,4]. In addition, genetic aspects explaining the individual susceptibility has been discussed [5]. In its severe forms, erosive tooth wear affects oral functions and aesthetics and may thereby have an impact on patient’s quality of life and overall well-being [6].

The global prevalence of erosive tooth wear ranges between 20 and 45% in the permanent dentition [7]. Studies from Greece indicate that erosive tooth wear is a major clinical problem with a prevalence that exceeds 50% in children and adolescents [8,9,10]. The diagnosis relies on a clinical examination in which the localisation and severity of the lesions are recorded. In order to monitor the progression of lesions and evaluate treatment effects, casted models and the use of validated scoring systems are helpful. Different indices are available, but none of them seem to have gained widespread acceptance in Europe [11,12].

A number of previous studies have investigated the awareness and knowledge concerning diagnosis and management of erosive tooth wear, unveiling a certain lack of knowledge among dentists and dental students [13,14,15,16,17,18,19,20,21,22]. Obviously, opinions, diagnostic measures, and treatment practices differ among dentists in various countries, possibly influenced by culture, education, tradition, and reimbursement models [13,16,17,18,19,20]. To the best of our knowledge, no such information is available from Greek dentists. This knowledge gap needs to be addressed in order to monitor the quality of care through customised educational activities and the promotion of regional and national guidelines. The aim of the present investigation was, therefore, to map the awareness, knowledge, and clinical management of erosive tooth wear among dentists practising in Greece. A secondary aim was to relate the outcome to the dentists’ age, clinical experience, and level of post-graduate education.

## 2. Material and Methods

### 2.1. Study Design

This cross-sectional study employed a case-based digital questionnaire, distributed to dentists practising in the greater Metropolitan area of Athens, Greece. The project was performed according to the Declaration of Helsinki’s (WMA 2013) ethical standards. The Ethics Committee, School of Dentistry, National and Kapodistrian University of Athens, Greece (Reg #N450), approved the research protocol. The participation was voluntary, and Google ensured the respondents’ anonymity.

### 2.2. Study Population

The eligible study population consisted of all active members of the Attica Dental Association (ADA) in Greece (n~5000). The sample size was calculated using the equation n = n0/Ν, where N was the total number of active members, and n0 = Z^2^_1−a/2_ p(1 − p)/e^2^. Given Z^2^_1−a/2_ = 1.64, the significance level a = 10%, *p* = 0.5, and a highest accepted error of e = 0.05, n0 was equal to 198. As N = 5000, n0/N equals 0.04, and as n < 0.05, the final sample size n = n0 and was therefore 198. To compensate for a 10% dropout rate, we invited a randomly selected group of 218 dentists to enter the survey. A member of the ADA, not involved in this study, performed the randomisation with the aid of computer-generated numbers.

### 2.3. Data Collection

Data were collected using a digital questionnaire (google forms, web application, google, docs.google.com/forms, google LLC, Mountain view, CA, USA), adopted from a similar questionnaire previously used for Finnish, Danish, Norwegian, and Icelandic dentists [16,17,18,19]. The questionnaire consisted of 24 questions divided into three sections: (i) registration and recording of erosive tooth wear, (ii) possible aetiological factors, and (iii) suggested treatment based on an illustrated clinical case. In the first section, we also collected information on the respondent’s gender, age, dental qualification, years of experience, and post-graduate education. We distributed the link to the survey to the selected dentists in June 2022, and it was open until September 2022. The respondents consented to participate by completing and submitting the questionnaire and maintained their anonymity throughout the whole process. Consequently, we were unable to issue any reminders to the non-responders.

We tested the applicability and repeatability of the questionnaire prior to the survey in a group of 35 selected dentists. We asked them to complete the survey and comment on its clarity. Minor revisions were made, and the final version showed high validity (k = 0.9) and repeatability (k = 0.8).

### 2.4. Data and Statistical Analysis

We transferred and collected the answers in an Excel spreadsheet (Microsoft Excel, Microsoft Corporation, Redmond, WA, USA) and used the IBM SPSS software (Version 21.0, SPSS Inc., Chicago, IL, USA) for the analyses. We used descriptive statistics to process the information, and answers were sub-analysed according to age, years of clinical experience, and level of dental education. We used chi-square and odds ratios and Student’s *t*-test for categorised and continuous data, respectively. Questions with more than one possible response were in some cases transferred into binary variables. The level of statistical significance was set at *p* < 0.05.

## 3. Results

### 3.1. Description of Respondents

A total of 200 dentists, 56 male (28%) and 144 female (72%), completed the questionnaire, giving a 91.7% response rate. A total of 50% were general dental practitioners (GDP), and 47% had a post-graduate degree in dental science. Seven percent had earned a PhD diploma. Overall, 80% were graduates of the National and Kapodistrian University of Athens, and 5% had graduated from a non-Greek dental school. Respondents worked in the private sector (82%) and in the central part of Athens (44%), and 35% had a mean working experience of ≥21 years.

### 3.2. Section i—Recording of Erosive Tooth Wear

Most respondents recorded the presence of erosive lesions (71%), of which 5% used a standardised and validated scoring system. Around one third of the respondents recorded only the location (tooth and surface) of the lesions. As few as 1% of the participants reported that they routinely captured digital photographs to keep track of the lesion progression. Five percent found it difficult to detect erosive tooth wear. We summarise the results according to years of experience and type of practice in Table 1. Dentists with fewer years in practice recorded erosive tooth wear more often by surface (*p* < 0.05), and those with any form of specialisation used a specific erosive index more often when compared with general dentists (*p* < 0.05).

### 3.3. Section ii—Aetiology

Table 2 presents the possible aetiological factors in relation to clinical experience and level of post-graduate education. The majority (>70%) claimed that they usually denoted the possible aetiological factors and 34% reported that they always assessed the dietary habits. The more experienced dentists and those in general practice preferred structured forms or templets, while dentists with fewer years of experience and specialists preferred a prospective food diary (*p* < 0.05). Measurements of the salivary secretion rate was very rare in all groups. In Table 3, we show the supposed aetiologic factors for erosive tooth wear according to the respondents. Extrinsic factors, such as fizzy soft drinks and acidic fruits/food, seemed to be the most common reason for erosions, while intrinsic factors (reflux and vomiting) were less frequent, albeit not uncommon.

### 3.4. Section iii—Treatment of Erosive Tooth Wear

More than 3/4 of the respondents (77%) managed patients with ETW in their own practice, 20% referred the patients to a specialist, and 3% referred them to a university clinic. The preventive measures were dominated by dietary advice (86%) followed by oral hygiene instructions (71%). Sixty percent prescribed fluoride mouth rinses and/or remineralising dental products. We show the odds ratio for the preferred treatment options, according to the respondents’ gender, age, and clinical experience, in Table 4. Younger and female dentists seemed to focus more on dietary advice compared with their elder and male peers (OR 1.2, *p* < 0.05). Similarly, respondents with a master’s degree in dental science tended to recommend fluoride mouth rinses and remineralising agents significantly more often when compared to general practitioners (OR 1.3, *p* < 0.05). It was also more common that the younger dentists referred their patients to a psychologist (OR 1.4, *p* < 0.05).

**Figure 1:**
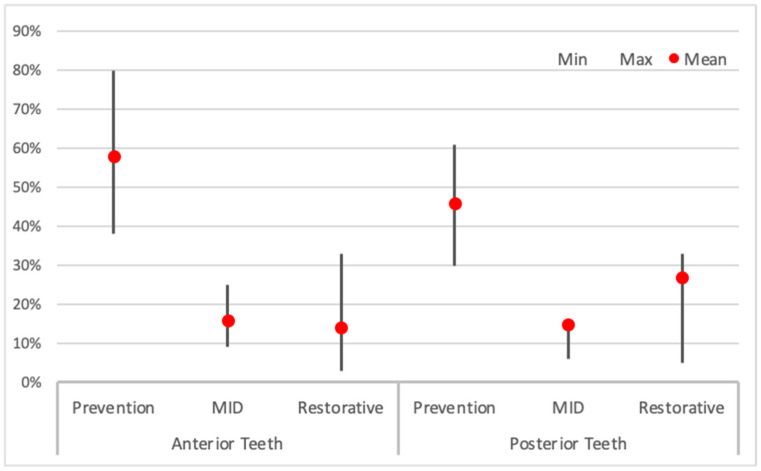
Management and treatment of erosive tooth wear in anterior and posterior teeth according to the respondents. The red dots indicate mean percentage for prevention, minimally invasive approaches (MID) and restorations. The vertical bars indicate the minimum and maximum values.

Based on the illustrated clinical case, we present the treatment of choice in relation to gender, age, years of practice, and specialisation in Table 5. The majority of the respondents preferred secondary prevention (dietary advice, oral hygiene, and fluoride exposure) as the main treatment option for all erosive tooth wear lesions, regardless of location (58% for lesions in the anterior region, and 46% for lesions in the posterior region (Figure 1)). Restorative treatment was more favoured in posterior than in anterior teeth (27% vs. 14%). The most frequently suggested technologies were resin infiltration, flowable resins/composites, and composite restorations. The use of composite onlays and metal crowns was more or less restricted to the lower molars.

## 4. Discussion

To our knowledge, this study is the first attempt to explore the knowledge and awareness of erosive tooth wear among Greek dentists. We took advantage of a previously validated digital questionnaire that allowed direct comparisons with findings from other countries in Europe and Jordan [16,17,18,19,20]. In contrast to the previous projects that suffered from low-to-very-low response rates, we adopted stratified randomised sampling model that gave few non-responders and, thereby, a low risk for selection and response bias. The novel and main findings were that Greek dentists largely presented similar awareness but shared diagnostic shortcomings including scoring and dietary and salivary analyses with their Scandinavian peers. Concerning the treatment decisions, most respondents had adopted a minimally invasive approach.

The majority of the respondents reported that they routinely recorded erosive tooth wear in the dental charts, a finding in accordance with previous studies in other countries around the world [17,18,19,20]. However, a minority (5%) used a detailed scoring system and similar findings were reported from Finland [16], Denmark [17], and Iceland [19]. In contrast, 50% of the Norwegian dentists used a specific scoring system, commonly at the tooth surface level [18]. It is strongly recommended that erosive tooth wear should be registered and monitored with the aid of a simple index, such as the Basic Erosive Wear Examination [11,12]. The difficulty with the different erosive indices is that they are subjective and potentially insensitive to small changes [12]. In spite of the shortcomings, the use of a scoring system introduces some level of standardisation of recording erosion progression and aid clinical decision-making [22]. The reason for the low compliance with a standardised scoring system is unclear. Dentists are used to routinely score and chart both caries and periodontal disease in a systematic way, and it seems unlikely that lack of knowledge would be the main barrier. A possible explanation could be that erosive tooth wear is asymptomatic in many cases and not the chief complaint [22]. Dentists may also perceive difficulties to distinguish between erosion, attrition, and abrasion [6,12]. We lack information on the type of dental records (digital or manual) that were used by the respondents. Thus, the “lack of space” reported by 15% of the respondents could not be further analysed. In any case, there is room for significant improvements in the clinical documentation of erosive tooth wear that call for educational and policy actions. The use of periodic clinical photographs and/or digital models should be encouraged.

Seventy percent of the participants reported that they tried to find the probable cause for the detected erosive tooth wear lesions, a percentage in harmony with previous observations [16,17,18,19,20,21]. Most respondents recognised carbonated drinks, fruit juice, and acidic food consumption as the most common aetiological factors of the lesions. This is in agreement with studies from Scandinavia [17,18,19], but geographic and cultural differences may exist. As an example, Al Ashtal and co-workers [13] reported that less than 50% of the dentists in Yemen reported that acidic drinks was a common aetiological factor for erosive tooth wear lesions. In Jordan, dentists thought that bruxism was the most common cause [20]. In the present study, gastroesopharyngeal reflux and vomiting were the major intrinsic causative factors for erosive tooth wear in the present project. It is therefore important that dentists refer the severe cases for pharmaceutical, surgical, and/or psychological treatment [23].

The methods for collecting data concerning dietary habits call for improvements. About one third of the respondents used a structured templet to record the intake of food and drinks, while younger clinicians and those with a specialisation tended to rely more on a diary logbook. Dietary assessment methods, including food records, food frequency questionnaires, 24 h recalls, and screening tools are time-consuming and far from perfect for collecting reliable information on the dietary intakes of individuals [15]. The benefit is that they send out an important motivational message to the patient that a behaviour change is important in order to arrest the ongoing erosion. Consequently, dentists must also be prepared to provide clear and personalised dietary recommendations and suggest less harmful food and drinks. In this context, dental health professionals have an obligation to actively educate public health peers and communities about dental erosion and motivate a change in the acidic beverage consumption behaviour [24].

In agreement with previous studies [16,18], very few respondents seemed to take advantage of salivary tests (secretion rate and buffer capacity) for diagnostic purposes. This could indicate a lack of knowledge on the protective role of saliva for erosive lesion development. One should, however, keep in mind that the questionnaire focused on young patients, and very few children and young adults display subnormal salivary secretion. Therefore, the low interest in salivary diagnostics unveiled here was partly explained and understandable.

Most respondents chose to treat patients with erosive tooth wear in their own clinic, and less than 25% referred them to a specialised practitioner or a university clinic. This figure was comparable with previous findings from Scandinavia [18,19]. It is possible that the option to refer patients are more due to the resources, availability, and reimbursement system rather than on the dentists’ level of knowledge. According to Mortensen and co-workers [17], Danish dentists referred the most severe and complex cases to hospitals and university clinics, in particular those that needed multi-disciplinary medical and dental care. For example, the association of GERD with erosive tooth wear is well established, and the prevention and management calls for such a skill-mix [22].

The finding that secondary prevention was the first treatment of choice for erosive tooth wear, regardless of location, was in line with previous findings from Europe [16,17,18,19,22] and current consensus statements recommendations [25]. The finding reflects an adequate understanding that prevention is better than a cure. The frequent recommendations to use topical fluorides and remineralising agents were expected, although the role of fluorides in the management of erosive tooth wear remain controversial [26]. Topical fluorides can help to remineralise enamel surfaces, and dental practitioners can support this process by applying topical fluoride varnish on teeth affected by erosion at recall appointments. In this context, products with stannous fluoride seem superior [27]. It is also important to make sure that patients use soft toothbrushes and toothpastes containing fluoride. Notably, a delayed brushing after the consumption of erosive foodstuffs or beverages does not seem capable of preventing erosive tooth wear [28]. Even for the somewhat more advanced lesions, the respondents seemed to favour the least invasive strategy, in particular for anterior teeth. This is in accordance with the minimally invasive approach to save hard tissue and decrease hypersensitivity [25,29]. Previous studies have shown that resin-based sealants can temporarily protect enamel and dentin from further erosion [30,31,32]. Composite fillings, indirect onlays, and crowns may be indicated for functional and/or aesthetic reasons [29]. In this survey, however, very few dentists suggested permanent restorations with the exception of the lower molars. This was in accordance with the European Consensus guidelines [25] that recommend minimally invasive techniques in the management of severe erosive tooth wear in order to keep other restorative options open in the future.

### Strengths and Limitations

The major strengths of this survey was the excellent response rate and that we stratified the sample to cover a range of characteristics among the dental practitioners. All dentists were from the greater metropolitan area of Athens, and we captured information from general practitioners and specialists with a different clinical experience. The randomly selected respondents were practicing in different areas and treated patients with different socioeconomic backgrounds, and this secured the internal validity. One can, however, always question the external validity, but as the outcome of this study was in harmony with findings from other countries, we consider the present study group to be representative for Greek and European peers. Nevertheless, surveys based on self-completed questions are always at certain risk of reporting bias. Respondents may tend to choose the most “expected” answers rather than reflecting their own practice. In real life, patients’ ability and willingness to pay will also influence the diagnostic and therapeutic alternatives to manage erosive tooth wear. We also lack information on whether the dentist used electronic or paper-based charts, which may have an impact on the quality of documentation and explain the “lack of space”. Another limitation was the inability to send reminders, but we consider the potential impact on sample representativeness as low due to the high response rate.

## 5. Conclusions

The majority of the dentists participating in this survey registered erosive tooth wear and its possible causes in the dental records. Preventive measures were well established, and the respondents seemed to have adopted a minimally invasive approach for the management of the condition in children and adolescents. Nevertheless, there was room for improvements in the case documentation; very few dentists used a standardised and validated scoring system in the charts, and dietary analysis and saliva diagnostics were not commonly taking into consideration establishing the etiopathogenesis of the condition. Thus, the results of this study indicate that clinicians’ ability to handle patients with erosive tooth wear needs to be partly updated through continuing educational courses that will offer updated treatment protocols based on the most current guidelines and focusing on patients’ needs and expectations.

## Figures and Tables

**Table 1:** Recording and scoring of erosive tooth wear in relation to the respondent’s clinical experience and type of practice. Values are expressed as percent, and figures in bold * denote a statistically significant difference.

	Years of Experience		Specialisation	
	<10 yrs	≥10 yrs	General Dental Practitioner	All Specialisations
	%	%	%	%
Do you record erosive tooth wear lesions?				
Yes (n = 141)	68	73	69	73
No (n = 59)	32	27	31	28
How do you evaluate them?				
No specific system (n = 119)	56	63	62	58
Location (n = 69)	36	33	36	33
Indices (n = 12)	8	4	2	10 *
How do you record them?				
Surface (n = 64)	40 *	24	26	38
Tooth (n = 69)	28	**41** *	31	38
Patient (n = 12)	3	9	10	2
Description (n = 46)	23	23	28	17
Photographs (n = 2)	2	0	1	1
Nothing (n = 8)	4	4	4	4
Why don’t you record them?				
No space (n = 29)	17	12	16	13
Difficulty in diagnosis (n = 4)	2	2	2	2
Time-consuming (n = 13)	6	7	7	6
Don’t know (n = 13)	6	7	7	6

* statistically significant *p* < 0.05 using chi-square or Fisher’s exact test.

**Table 2:** Number and proportion of the responses regarding recording of aetiological factors for erosive tooth wear in relation to the respondent’s clinical experience and type of practice. Values are expressed as percent and figures in bold * denote a statistically significant difference.

	Years of Experience		Specialisation	
	<10 yrs	≥10 yrs	General Dental Practitioners	All Specialisations
	%	%	%	%
When you see an erosive lesion do you record possible etiological factors				
Usually (n = 150)	76	74	71	78
Occasionally (n = 43)	21	22	26	18
Rarely (n = 3)	2	1	2	1
Don’t know (n = 4)	1	3	1	3
In patients with erosive tooth wear lesions do you assess their dietary habits				
Never (n = 30)	11	19	15	15
Sometimes (n = 102)	53	49	59	44
Always (n = 68)	36	32	26	42
How do you assess the patient’s dietary habits				
Structured questionnaires (n = 121)	45	**76** *	**65** *	54
Diaries (n = 47)	**39** *	8	13	**33** *
Simple questions (n = 35)	16	19	21	14
In patients with erosive tooth wear lesions do you evaluate their salivary secretion				
Never (n = 153)	81	72	79	74
Sometime (n = 39)	14	25	18	22
Always (n = 7)	4	3	3	4

* statistical significance *p* < 0.05 using chi-square or Fisher’s exact test.

**Table 3:** Frequency of the perceived possible aetiological factors of erosive tooth wear according to the respondents.

	*Fizzy Drinks*	*Energy Drinks*	*Acidic Beverages*	*Gastroesopharyngeal Reflux*	*Vomiting*
*Often*	67%	38%	56%	45%	35%
*Sometimes*	28%	44%	37%	47%	51%
*Never*	4%	14%	6%	5%	11%
*Don’t know*	1%	4%	1%	3%	4%

**Table 4:** Odds ratio (confidence interval) for reporting specific treatment options, according to clinicians’ gender, age, and clinical experience.

	*Dietary Advice* *(86%)*			*Oral Hygiene Instructions* *(71%)*			*Fluoride Mouth Rinse* *(60%)*			*Chlorhexidine Mouthwash* *(3%)*			*Remineralisation Products* *(56%)*			*Referral to Specialised Dentist* *(27%)*			*Referral to a Psychologist* *(37%)*		
	***Odds Ratio***	***95% CI***	***p-Value***	***Odds Ratio***	***95% CI***	***p-*** ***Value***	***Odds Ratio***	***95% CI***	***p-*** ***Value***	***Odds Ratio***	***95% CI***	***p-*** ***Value***	***Odds Ratio***	***95% CI***	***p-Value***	***Odds Ratio***	***95% CI***	***p-Value***	***Odds Ratio***	***95% CI***	***p-Value***
**Gender**																					
Female	1.2	1–1.4	**0.01**	1.1	0.9–1.4	0.14	1	0.8–1.2	0.48	1.3	0.9–1.2	0.06	1	0.8–1.3	0.53	1.15	0.9–1.4	0.1	1.2	0.8–1.9	0.23
Male	Ref			Ref			Ref			Ref			Ref			Ref			Ref		
**Age**																					
<40 yrs	1.2	0.9–1.2	**0.05**	1.2	0.9–1.4	**0.05**	1.2	0.9–1.5	0.09	1	0.2–4.9	**0.64**	1	0.7–1.1	0.12	1.6	0.9–2.5	0.04	1.4	0.9–2.1	0.04
≥40 yrs	Ref			Ref			Ref.			Ref			Ref			Ref			Ref		
**Years of experience**																					
Up to 10 yrs	1.2	1–1.3	**0.03**	1.1	0.9–1.4	**0.11**	1.2	0.9–1.5	0.11	1.3	0.3–6.2	**0.53**	0.8	0.6–1.1	**0.12**	1.4	0.8–2.1	0.14	1.4	0.9–1.9	0.06
≥10 yrs	Ref			Ref			Ref			Ref			Ref			Ref			Ref		
**Specialisation**																					
Yes	1.1	0.9–1.2	**0.21**	1.1	0.8–1.3	0.34	1.3	1–1.6	0.02	1	0.2–4.9	**0.65**	1.5	1.1–2	**0.01**	1.3	1.1–1.5	**0.02**	1	0.7–1.4	0.46
No	Ref			Ref			Ref			Ref			Ref			Ref			Ref		

**Table 5:** Case-based treatment proposed for erosive tooth wear in upper anterior teeth and upper and lower molars.

	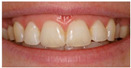		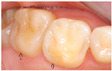		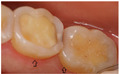	
	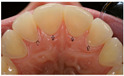		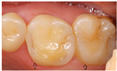		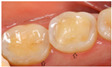	
***Treatment Proposed***	***Upper Central Incisor*** ***n (%)***	***Upper Lateral Incisor*** ***n (%)***	***Upper First Molar*** ***n (%)***	***Upper Second Molar*** ***n (%)***	***Lower First Molar*** ***n (%)***	***Lower Second Molar*** ***n (%)***
*Dietary advice*	159 (80)	154 (77)	137 (69)	124 (62)	122 (61)	122 (61)
*Fluoride mouth rinse*	125 (63)	120 (60)	104 (52)	93 (47)	92 (46)	91 (46)
*Fluoride varnish*	100 (50)	97 (49)	91 (46)	76 (38)	62 (31)	59 (30)
*Application of adhesive*	19 (10)	21 (11)	20 (10)	18 (9)	11 (6)	10 (5)
*Resin infiltration*	39 (20)	38 (19)	23 (12)	20 (10)	21 (11)	16 (8)
*Application of flowable resin*	47 (24)	50 (25)	40 (20)	35 (18)	26 (13)	27 (14)
*Glass ionomer cement*	9 (5)	10 (5)	15 (8)	13 (7)	11 (6)	10 (5)
*Composite restoration*	57 (29)	55 (28)	65 (33)	57 (29)	65 (33)	62 (31)
*Onlay*	19 (10)	20 (10)	28 (14)	23 (12)	39 (20)	44 (22)
*Metal crown*	5 (3)	6 (3)	24 (12)	22 (11)	33 (17)	34 (17)

## Data Availability

The datasets used and/or analysed during the current study are available from the corresponding author on reasonable request.
